# Biophysical Properties as Determinants for Soil Organic Carbon and Total Nitrogen in Grassland Salinization

**DOI:** 10.1371/journal.pone.0054827

**Published:** 2013-01-23

**Authors:** Chengchen Pan, Halin Zhao, Xueyong Zhao, Huibang Han, Yan Wang, Jin Li

**Affiliations:** Cold and Arid Regions Environmental and Engineering Research Institute, Chinese Academy of Sciences, Lanzhou, China; Roehampton University, United Kingdom

## Abstract

Grassland salinization causes considerable changes to soil and vegetation, which can lead to changes in soil organic carbon (C) and total nitrogen (N). These changes have complex causal relationships. A significant correlation between soil organic C and total N and any soil or vegetation property does not necessarily imply a significant direct effect of the property on soil organic C and total N. In this study, a field survey was conducted to investigate the changes in soil organic C and total N in grassland along a salinity gradient in Hexi corridor, China, and the direct and indirect effects of soil and vegetation properties on both stocks were quantified using a path analysis approach. Significant decrease in soil organic C and total N contents were observed with increasing salinity. Both had significant positive correlations with the Normalized Difference Vegetation Index (NDVI), soil water, and fine particles (silt+clay) content (*p*<0.01) and significant negative correlations with soil EC, and sand content (*p*<0.01). NDVI, fine particles content and soil water content had positive direct effects on soil organic C and total N stocks. Soil EC affected soil organic C and total N stocks mainly through its indirect negative effect on NDVI, soil texture, and water content. NDVI, soil texture, and moisture also indirectly affected soil organic C and total N stocks via changes in each other. These indirect effects augmented each other, although in some cases indirect effects worked in opposing directions.

## Introduction

Soils play an important role in the carbon (C) and nitrogen (N) cycles of the earth [1]. At the global scale, they contain ∼75% of the C and >90% of the N in the terrestrial biosphere [1]. The cycles of these two elements often play an essential role in determining the physical and chemical characteristics of a soil and therefore in determining its fertility, and are pivotal in determining responses of an ecosystem to environmental stresses. C and N also play major roles in contributing to the atmospheric concentrations of other greenhouse gases, such as CH_4_ and N_2_O [2], [3]. Understanding changes in soil C and N contents is not only critical in determining soil quality (e.g. fertility, water holding capacity) and ecosystem productivity, but also in quantifying the influence of changing rates of C and N cycles and storage on global climate change. The frequency and potential consequences of changes in soil C and N contents have been intensively explored in many ecosystems during the last decades [4]–[7]. Soil organic C stocks, for example, may be lower in saline soils due to poor plant growth and thus lower C inputs. However, lower decomposition rates in these soils could counteract this effect, leading to similar or even higher soil organic C stocks than found in low-salinity soils, despite lower inputs [8]. Because salinity may affect soil C and N contents compared to other soils, a better knowledge of soil organic C and total N in salt-affected soils is needed.

Salinization, as one of the most serious types of land degradation, has become a major concern throughout the world [9], [10]. Salt-affected soils occur mainly in arid or semiarid regions, where evaporation greatly exceeds precipitation and salts dissolved in the ground water reach and accumulate at the soil surface through capillary movement [10]. Worldwide, approximately 932 million ha are estimated to be salt affected [11]. Salt-affected soils cover about 10% of the total surface of dry land [11], and this situation is getting worse in many parts of the world [12]. According to the database of China’s second national soil survey, soil salinization affects an estimated 35 million ha, of which 29.3 million ha are salinity affected grasslands. Saline condition was closely related to the soil organic matter accumulation and decomposition [13], [14] which would, therefore, affect soil organic C and N cycling [15].

A number of biotic and abiotic variables have been reported to be closely related to soil C and N. Such variables include climate, vegetation, topography, intrinsic soil properties, land use and management practices, etc. [16], [17]. However, the inter-correlations among these variables demand caution in interpreting correlations between soil organic C and total N and these variables. Thus, a strong correlation of a variable with soil organic C and total N may not necessarily imply the direct effect of the variable on organic C and total N of the soil, but may be due to the indirect effect of some other variables. As such, simple correlation analysis may not be sufficient in evaluating the direct influence of these variables on soil organic C and total N stocks. An integrative approach combining these variables with correlation and path analyses is needed. However, such an integrative research is still limited. Path analysis was developed to organize and present relationships between dependent and independent variables, thus decomposing the relationships into different pieces for interpretation of effects [18]. It decomposes the source of the correlation among variables and, thus, partitions the correlation between dependent and independent variables into direct and indirect effects [18]. While path analysis has been employed in soil studies to investigate the cause and effect of soil properties on heavy metal adsorption, P retention [19], [20], its application to soil organic C and total N to soil and vegetation properties was a new development for grassland salinization.

Because of the close relationships between soil and vegetation properties and soil organic C and total N contents, efforts have been made to predict soil organic C and total N contents from these variables using various combinations. It is important to determine soil and vegetation properties that have significant direct influence on soil organic C and total N. This information enables the estimation of soil organic C and total N using appropriate soil and vegetation properties contained in (i) routine soil survey databases (e.g. EC, soil water content, %Sand, %silt and %fine particles); and (ii) routine vegetation database such as the Normalized Difference Vegetation Index (NDVI) and ecosystem productivity. Therefore, the objectives of this study were: 1) to examine changes in soil and vegetation properties and soil organic C and total N contents in the process of grassland salinization; and (2) to examine the direct and indirect effects of vegetation and soil properties on soil organic C and total N stocks.

## Materials and Methods

### Ethics Statement

Linze Grassland Ecological Test Station (100°02′E, 39°15′N) is a department of Lanzhou University. This study was approved by the College of Pastoral Agriculture Science and Technology, Lanzhou University.

### Study Area

This experiment was conducted in Linze Grassland Ecological Test Station (100°02′E, 39°15′N) in the middle reaches of the Hexi Corridor region at an average altitude of 1400 m above sea level in Gansu Province, PR China. The station is located at the southern edge of the alluvial fans of the Heihe River. The Heihe River runs down from the Qilian Mountains; melt water from glaciers and snow cover is the principal source of surface runoff. The ground water table is at a depth of 0–2.5 m, with periodic rises during the year. The climate in this region is characterized as temperate continental arid monsoon, dry and hot in the summer and cold in the winter. The annual mean precipitation is 121.5 mm, of which 61% is received in summer and autumn; the annual mean evaporation is over 2,338 mm. Annual mean temperature is 7.1°C, with the absolute maximum up to 38°C and minimum –28°C. Salt-affected grasslands are estimated to encompass 1.4 million ha, accounting for 79% of the total salt-affected soils in Hexi corridor, China [21]. The soils in this region are identified as salinized meadow soils and salinity soils which are mainly sulphate salt, and which have an alkaline pH ranging from 8.40 to 8.68 with no significant difference among types [22].

### Experimental Design

For the present work, a space-for-time substitution approach was used [23]. According to the classification of salinization types and degrees [24], four types of salinized grasslands were selected including lightly (200–400 mS/m), moderately (400–800 mS/m), heavily (800–1600 mS/m), and severely (>1600 mS/m) salinized grasslands. The common species found among the different salinized grasslands include *Phragmites communis* and *Legmus dasystachys*. Species unique to specific grassland types are *Kalidium gracile* to the moderately salinized grassland; *Achnetherum splendens* to the heavily salinized grassland; and *Nitraria tangutorum* to the severely salinized grassland [22]. For each of the four types of salinized grassland, three survey sites (10 m×10 m) were established for measurements.

Five soil samples were collected and combined at each plot from 0–30 cm depth. Soil samples were placed in sealed plastic bags and brought to the laboratory. Soil bulk density was determined at each site using a core sampler (stainless steel cylinder with a volume of 100 cm^3^) at three depths (0–10, 10–20 and 20–30 cm), with five replicates. All samples were collected in August 2011.

### Data Collection and Analysis

Landsat TM 30-m resolution imagery for the studied plots was acquired during a cloud-free day: August 1 in 2011. Using bands from the red (*red*) and near-infrared (*NIR*), a floating point raster was also generated for each scene containing values for NDVI [25]. NDVI values were calculated using the following formula [26]:
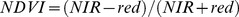
(1)where *NIR* is the reflectance signal in the near-infrared (band 4 in Landsat TM) and *red* is reflectance in the red band (band 3 in Landsat TM)

In the laboratory, each soil sample was thoroughly sieved to 2 mm to remove roots and incorporated litter, air-dried, and divided into two parts. One part was used first for determining particle size distribution and electrical conductivity (EC). The second part was further sieved to 0.25 mm and used for determining average organic C and total N. Soil particle size distribution was determined by the pipette method in a sedimentation cylinder, using Na-hexamethaphosphate as the dispersing agent [27]. EC was determined in a 1∶5 soil–water extract. Soil organic C was measured by the K_2_Cr_2_O_7_–H_2_SO_4_ oxidation method of Walkey and Black [28]; total N by the Kjeldahl procedure (UDK 140 Automatic Steam Distilling Unit, Automatic Titroline 96, Italy) [29]. The total amount of organic C and N in each soil was calculated by the following equation [2]:
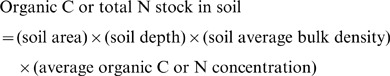
(2)


### Statistical Analysis

Because data were not normally distributed, the Kruskal-Wallis test was used to determine the differences among the four grassland types. Pearson correlation analysis was used to study the relationships between the corresponding variables. Aside from this, path analysis was performed to investigate the direct and indirect effects of vegetation and soil properties on soil organic C and total N stocks [18]–[20]. Prior to the path analysis, all variables were log_e_(N+1) transformed for normality. All data were analyzed using DPS software.

## Results

### Changes in NDVI and Soil Physical Properties

As shown in Table 1, with salinization development, soil EC and sand (2∼0.05 mm) content increased significantly, while NDVI and soil water decreased significantly (*P*<0.05). Although bulk density did tend to increase with salinity, it was not as sensitive to the different classes as were NDVI and soil parameters. No changes in bulk density were found among the remaining three grassland types except for the lightly salinized grassland.

**Table 1:** Vegetation and physical properties in grasslands as affected by salinization.

Degree of salinization	EC (mS/m)	NDVI	SWC (%)	Sand* (%)	Silt (%)	Clay (%)	Bulk density (g/cm^3^)
Lightly	320.2±23.7d	0.407±0.043a	36.5±1.9a	14.2±1.1c	85.4±0.9a	0.4±0.3a	1.07b
Moderately	477.2±18.6c	0.310±0.082a	26.7±0.3b	26.2±1.9b	73.4±1.8ab	0.4±0.1a	1.25a
Heavily	906.1±52.6b	0.063±0.006b	23.8±0.6c	29.2±1.0b	70.8±1.0b	0b	1.25a
Severely	1760.0±125.9a	0.033±0.012c	17.2±0.5d	33.3±0.2a	66.7±0.2b	0b	1.25a

Values are means±SE. Values with the same letters within columns are not significantly different at *P*<0.05. EC, electrical conductivity; SWC, Soil water content. *Sand, 2∼0.05 mm; Silt, 0.05∼0.002 mm; Clay, <0.002 mm.

Compared to the lightly salinized grassland, soil EC and sand content increased by 449.7% and 136.4% (Table 1), and NDVI, soil water, silt and clay contents decreased by 91.9%, 52.9%, 21.9% and 100% in the severely salinized grassland, respectively. Generally, soil texture becomes coarser, and vegetative cover sparser with the development of grassland salinization.

### Changes in Soil Organic C and Total N Concentrations and Stocks

Soil organic C and total N concentrations decreased significantly (*P*<0.05) with increasing salinity (Table 2). Compared to the lightly salinized grassland, organic C decreased by 76.0%, from 14.60 to 3.50 g/kg soil, and total N decreased by 73.6%, from 1.40 g/kg to 0.37 g/kg in the severely salinized grassland. The magnitude of decrease differed among salinization stages. The decrease in organic C and total N concentrations was greatest from the lightly to the moderately salinized stage, reduced by 40.9%, 35.7% from the lightly to the moderately salinized grassland, respectively. C/N ratios decreased with salinization development (except for the severely salinized grassland) but were not significant.

**Table 2:** Changes in soil organic C and total N contents in grasslands as affected by salinization.

Degree of salinization	Concentrations (g/kg soil)		C/N	Stocks (kg/m^2^)	
	Organic C	Total N		Organic C	Total N
Lightly	14.60±1.01a	1.40±0.05a	10.43±0.20a	4.70±0.21a	0.45±0.03a
Moderately	8.63±0.77b	0.90±0.08b	9.57±0.09b	3.24±0.26b	0.34±0.03b
Heavily	4.67±0.82c	0.51±0.06c	9.03±0.56b	1.73±0.28c	0.19±0.02c
Severely	3.50±0.15c	0.37±0.02d	9.57±0.28b	1.31±0.03c	0.14±0.01d

Values are means±SE. Values with the same letters within columns are not significantly different at P<0.05.

As shown in Table 2, the organic C and total N stocks per unit area (0–30 cm depth) decreased significantly (*P*<0.05) with the progression of salinization. Compared to the lightly salinized grassland, even in the moderately salinized grassland the loss of organic C and N reached 1.46 kg/m^2^ and 0.11 kg/m^2^, respectively, while in the severely salinized grassland losses were 3.39 kg/m^2^ and 0.31 kg/m^2^.

### Relationships between Soil Organic C and Total N Stocks and Environmental Variables

Table 3 shows the correlations among organic C stock, total N stock and various environmental variables, including vegetation (NDVI) and soil properties (soil water content, EC, and texture). Soil total N stock correlated closely with organic C stock. Organic C and total N stocks had a significant positive correlation with four of the six variables (NDVI, soil water, silt and clay content), whereas a significant negative correlation with two (soil EC and sand content).

**Table 3:** Pearson correlation coefficients among parameters and organic C and total N stocks.

Items	C stock	N stock
Sand (%)	−0.926**	−0.910**
Silt (%)	0.892**	0.873**
Clay (%)	0.720**	0.776**
EC	−0.793**	−0.822**
Soil water content (%)	0.933**	0.927**
NDVI	0.892**	0.882**
N stock	0.992**	

** *P*<0.01.

Path analysis showed differential direct and indirect effects of vegetation (NDVI) and soil properties on soil organic C and total N stocks (Table 4). NDVI and soil fine particles (clay+silt) content had far greater direct effects on soil organic C stock (path coefficients: 0.551, 0.400) than did soil water content and EC (path coefficients: 0.235, 0.160). Path analysis also supported the hypotheses that soil EC affected soil organic C stock indirectly through its negative effect on NDVI, soil water content and fine particles content. Soil water content had an effect on soil organic C stock indirectly through its positive effect on NDVI and fine particles content. NDVI had an effect on soil organic C stock indirectly through its positive effect on fine particles content. Fine soil particles had an effect on soil organic C stock indirectly through its positive effect on NDVI and soil water content (Table 4). Although there were some differences in certain aspects, patterns were similar between soil organic C stock and total N stock (Table 4).

**Table 4:** Path analysis to test for the direct and indirect effects of vegetation and soil properties on soil organic C and total N stocks in grassland salinization.

Independent variable	Direct effects	Indirect effects			
		*x*_1_→*Y*	*x*_2_→*Y*	*x*_3_→*Y*	*x*_4_→*Y*
	Soil organic C (*Y*)				
EC* (*x*_1_)	0.160	–	−0.494	−0.219	−0.345
NDVI (*x*_2_)	0.551	−0.144	–	0.193	0.311
Soil water content (%) (*x*_3_)	0.235	−0.149	0.452	–	0.372
Fine particles (%) (*x*_4_)	0.400	−0.139	0.429	0.219	–
	Total N (*Y*)				
EC* (*x*_1_)	0.185	–	−0.453	−0.316	−0.314
NDVI (*x*_2_)	0.505	−0.166	–	0.279	0.283
Soil water content (%) (*x*_3_)	0.340	−0.172	0.414	–	0.337
Fine particles (%) (*x*_4_)	0.363	−0.161	0.394	0.316	–

* EC, electrical conductivity.

## Discussion

### Changes in Soil and Vegetation Properties

NDVI is a commonly used and easily calculated satellite image-based proxy for vegetation productivity [26], [30]. The results of this study showed that with soil salinization development, NDVI decreased significantly (Table 5) and concomitant bare surfaces increased significantly with soil salinization development (i.e. reduced vegetation cover or productivity and enhanced soil erodibility), indicating that a key variable associated with increasing salinity is reduced vegetation productivity (represented as NDVI in this case). There are two possible ways by which enhanced salinity has a significant influence on NDVI. First, in our study soil water content decreased significantly because ground water table decreased (data not shown) with salinization development, leading to lower soil water availability. Plant access to soil water is decreased with the osmotic potential of the soil solution. As a result, there may be a decrease in vegetation productivity on the ground. Second, high salt concentrations might affect plant survival through changes in ion toxicities and deficiencies [31]. Therefore, the observed among-type variation in NDVI can be explained by combined differences in both osmotic potential and ion toxicities among the four grassland types.

**Table 5:** Matrix correlation among soil and vegetation properties in grasslands as affected by salinization.

	EC	NDVI	SWC (%)	Sand (%)	Silt (%)
NDVI	−0.78**				
SWC (%)	−0.84**	0.80**			
Sand (%)	0.76**	−0.76**	−0.94**		
Silt (%)	−0.73**	0.72**	0.88**	−0.97**	
Clay (%)	−0.57*	0.51	0.65*	−0.67*	0.63*

EC, electrical conductivity; SWC, Soil water content.

* *P*<0.05,

** *P*<0.01.

Additionally, soil physical properties can be affected. A soil’s ability to percolate water (permeability and infiltration), how much water the soil can store (available water holding capacity), and the soil’s ability to adsorb or desorb chemical ions (exchange capacity) properties are all strongly related to soil texture [32]. For example, sandy soils have relatively lower water holding capacities and are faster to drain because of their bigger pore diameters, while clay soils retain more water and are slower to drain. In this study, fine particles (silt+clay) decreased significantly and concomitant sand increased significantly with increasing salinity. This maybe attribute to the enhancement in wind and water erosion with increasing salinity. The decrease in fine particles caused progressive coarsening, available water capacity decreasing, which would have a negative and potentially lethal effect on plants (Table 5).

Soil and vegetation resources are closely correlated, soils influence plants, and plants affect the characteristics of soils inversely [33]. The presence of vegetation aided in maintaining several important soil properties in contrast with results of those soil properties in the heavily and severely salinized grasslands. For example, the presence of vegetation in the lightly and moderately salinized grasslands improved the soil’s available water-holding capacity (data not shown), and reduced soil erosion. Vegetation properties also affect ecosystem processes such as litter production and turnover [34], resulting in different organic C and total N contents in soil [35], [36]. Data from the present study showed that the low soil organic C and total N stocks found in the severely salinized grassland were nearly two times lower than that found in the lightly salinized grassland, indicating that soil organic C and total N stocks were significantly related to the degree of salinization. These results are consistent with findings from other studies suggesting that the soil organic C and total N storage decreased substantially with grassland degradation [37].

### The Relative Importance of Soil and Vegetation Properties Determinants to Soil Organic C and Total N Contents

Soil organic C and total N contents were closely correlated to soil texture, water content, EC and vegetation productivity (Table 3), which had been reported separately in many studies [15], [38]–[40]. Significant correlations were also observed among most of the soil and vegetation properties in the current study (Table 5). Understanding the factors that influence soil organic C and total N contents in salinized grasslands can help to discern mechanisms affecting them, which is critical in environmental management. Path analysis was considered to be a powerful analytical tool to determine the potential interlinked influences of biophysical properties in explaining soil organic C and total N contents in salinized grasslands. These results support the hypotheses that soil conditions (i.e., soil EC, texture and moisture), and vegetation property may influence soil organic C and total N stocks. This suggests that the effects of soil and vegetation properties are complementary.

The direct effects of biophysical properties found in path analysis were the positive effects of NDVI, fine particles (%) and soil water content (%) on soil organic C and total N stocks. Plant traits clearly affect soil organic C and total N cycling [41]. Plant growth is the primary source of soil organic C, and the strong positive correlation between vegetation productivity and soil organic C was widely reported [39], [40]. Inputs to soil may be greater in more diverse communities because high plant diversity may enhance soil fertility or improve ecosystem stability and productivity [41]. Vegetation degradation is one of the most important consequences of land salinization in natural grasslands. Vegetation productivity and plant diversity (data not shown) decreased significantly with increasing salinity in this study. These dereases had the potential to decrease C inputs into the soils.

Soil texture also has a strong effect on the soil’s capacity to accrue C; numerous studies in rangelands [42] have shown that C stocks are positively related to the soil’s clay or fine particles contents, so changes in soil’s clay or fine particles contents could markedly affect soil organic C stocks. Fine particles affect soil organic C stocks through influencing the formation of clay–humus complexes that stabilize and retain more organic matter [43], [44].

Soil moisture, as an abiotic driver of soil organic matter dynamics, is well studied [41]. The temperature sensitivity of soil organic C decomposition might be affected by soil moisture. However, no consensus exists regarding soil moisture and the temperature sensitivity of soil organic C decomposition [45]. For example, for soils collected from shallow, xeric uplands at a mesic grassland, temperature sensitivity was greatest at intermediate soil moisture, but for soils collected from the deeper, mesic lowlands, temperature sensitivity increased with increasing soil moisture [45]. Moreover, microbial activity is regulated by soil moisture, which protects organic matter from decomposition by high- and low-moisture conditions [41]. In this study, soil water content directly and positively influenced soil organic C content. This result is in accordance with other studies stating that the influence of soil water content on soil organic C content is positive [46], [47].

Path analysis also showed that, in the salinized grasslands, the main influence of soil EC on soil organic C and total N stocks was indirect through its negative effect on NDVI, soil texture, and water content. Even though simple correlation analysis indicated that the correlation between soil organic C and total N stocks and soil EC was significant, the direct effect of soil EC was not significant. This further shows that simple correlation analysis is not enough to describe the relationship between soil organic C and total N stocks and soil properties. Meanwhile, the interaction among soil texture, moisture and vegetation showed indirect effects on soil organic C and total N stocks. For example, vegetation property affected soil organic C and total N stocks indirectly through its positive effect on fine particles, while it affected fine particles indirectly through its positive effect on NDVI and soil water content (Table 4). These indirect effects augmented each other (primarily yielding positive effects), although in some cases indirect effects worked in opposing directions.

The observed patterns in soil organic C and total N stocks in salinized grasslands depend on complex relationships among vegetation and soil factors whose influence is difficult to isolate. Although the combination of good statistical technique with previous knowledge of the system and new ecological theories allows us to better understand this ecosystem [48], experimental measurements are needed for causal evidence of the hypothesis proposed in this study. The direct and indirect effects of biophysical factors on soil organic C and total N will be due to independent variables not measured.
